# Evaluation of Interexaminer Reliability in Visual Shade Matching: A Comparative Analysis of Optilume Trueshade and Conventional Lighting for Permanent Anterior Teeth

**DOI:** 10.7759/cureus.108421

**Published:** 2026-05-07

**Authors:** Omar F Alkhalifah, Shahzad Ali Shah, Sabahat Ullah Khan Tareen, Muhammad Zubair Ahmad

**Affiliations:** 1 Department of Conservative Dental Sciences, College of Dentistry, Qassim University, Buraydah, SAU

**Keywords:** anterior teeth, color perception, dental esthetics, tooth color, tooth shade matching

## Abstract

Background: Achieving a lifelike color match for anterior restorations is a persistent challenge because tooth appearance is heavily influenced by illumination and observer-dependent choices. While visual matching with VITA-type guides remains the most common global approach, its reproducibility often drops under varying ambient light.

Objectives: The study sought to determine whether using a light-correcting device (Optilume Trueshade, ~5,500 K; Optident Dental Products, Ilkley, West Yorkshire) meaningfully enhances interexaminer reliability when selecting shades for maxillary anterior teeth with the VITAPAN Classical A1-D4 shade guide (VITA Zahnfabrik, Germany) compared with conventional clinical lighting.

Methodology: In this cross-sectional study, 50 adults (18-40 years) with vital maxillary incisors were randomized into Group A (conventional lighting) and Group B (Optilume Trueshade). Three blinded restorative dentists independently selected shades on the middle third of a randomly selected reference tooth. Reliability was assessed using Cronbach’s alpha, while shade selection differences were analyzed via Kruskal-Wallis and post hoc tests.

Results: Internal consistency was excellent, with both systems achieving a Cronbach’s alpha of 0.91 and an overall study reliability of 0.94. For teeth 11, 12, and 22, no statistically significant differences in shade selection were found between operators. A significant discrepancy was observed for tooth 21 (p = 0.002); however, post hoc analysis revealed it was operator-specific rather than a flaw in the lighting system.

Conclusion: Both the VITAPAN Classical guide and the Optilume Trueshade system are highly reliable tools for shade matching in restorative dentistry. Individual human factors, such as fatigue and physiological perception, remain significant variables, highlighting the need for periodic calibration and strengthened operator training.

## Introduction

Achieving a lifelike color match for anterior restorations remains a persistent challenge because tooth color is influenced by enamel/dentin translucency, spatial nonuniformity across the crown, and critical illumination. Visual shade selection using the VITAPAN Classical A1-D4 shade guide (VITA Zahnfabrik, Germany) remains the most common approach worldwide, but its reproducibility declines when ambient light varies in color temperature and spectral quality, leading to metamerism and observer-dependent choices [1]. Contemporary guidance, therefore, recommends daylight-simulating light (≈5,500-6,500 K) with a high color-rendering index (CRI ≥90) to minimize perceptual error and improve consistency [2].

Recent comparative studies highlight both the promise and the limits of newer digital approaches. Clinical investigations and meta-analyses show that intraoral scanners’ shade functions and spectrophotometers do not always agree with visual methods, and agreement between instruments themselves is only slight to moderate; repeatability is often better within a given device than between methods. These findings suggest that optimizing visual workflows (e.g., with standardized lighting) remains clinically relevant rather than being supplanted outright by digital tools [3-5].

Another contributor to inconsistency is the coverage error inherent to popular shade guides (including VITA Classical A1-D4) when mapped onto natural tooth color spaces; some areas of the in vivo color gamut remain underrepresented by available tabs. This structural limitation can compound perceptual variability under suboptimal lighting, strengthening the case for environmental standardization during visual matching [6].

Light-correcting devices have, therefore, been proposed as a low-complexity, chairside strategy to deliver controlled, daylight-like illumination for shade selection. Emerging clinical evidence indicates that such 5,500 K, high-CRI corrective lights can improve the reliability of visual shade matching among clinicians and students compared with routine operatory lighting, an effect observed across experience levels and tooth positions [1,7].

Against this backdrop, the present study focuses on whether using a light-correcting device (~5,500 K) meaningfully enhances interexaminer reliability when selecting shades with the VITA Classical A1-D4 guide for maxillary anterior teeth, compared with conventional clinic lighting. Demonstrating a reliability gain under standardized illumination would support a pragmatic protocol that can be adopted broadly in everyday practice without requiring specialized digital hardware.

## Materials and methods

Study design

A cross-sectional comparative study was conducted at the College of Dentistry, Qassim University, Saudi Arabia, following approval from the institutional ethical review committee (approval no. 24-84-17). The study aimed to evaluate interexaminer reliability in visual shade matching under two lighting conditions.

**Figure 1:**
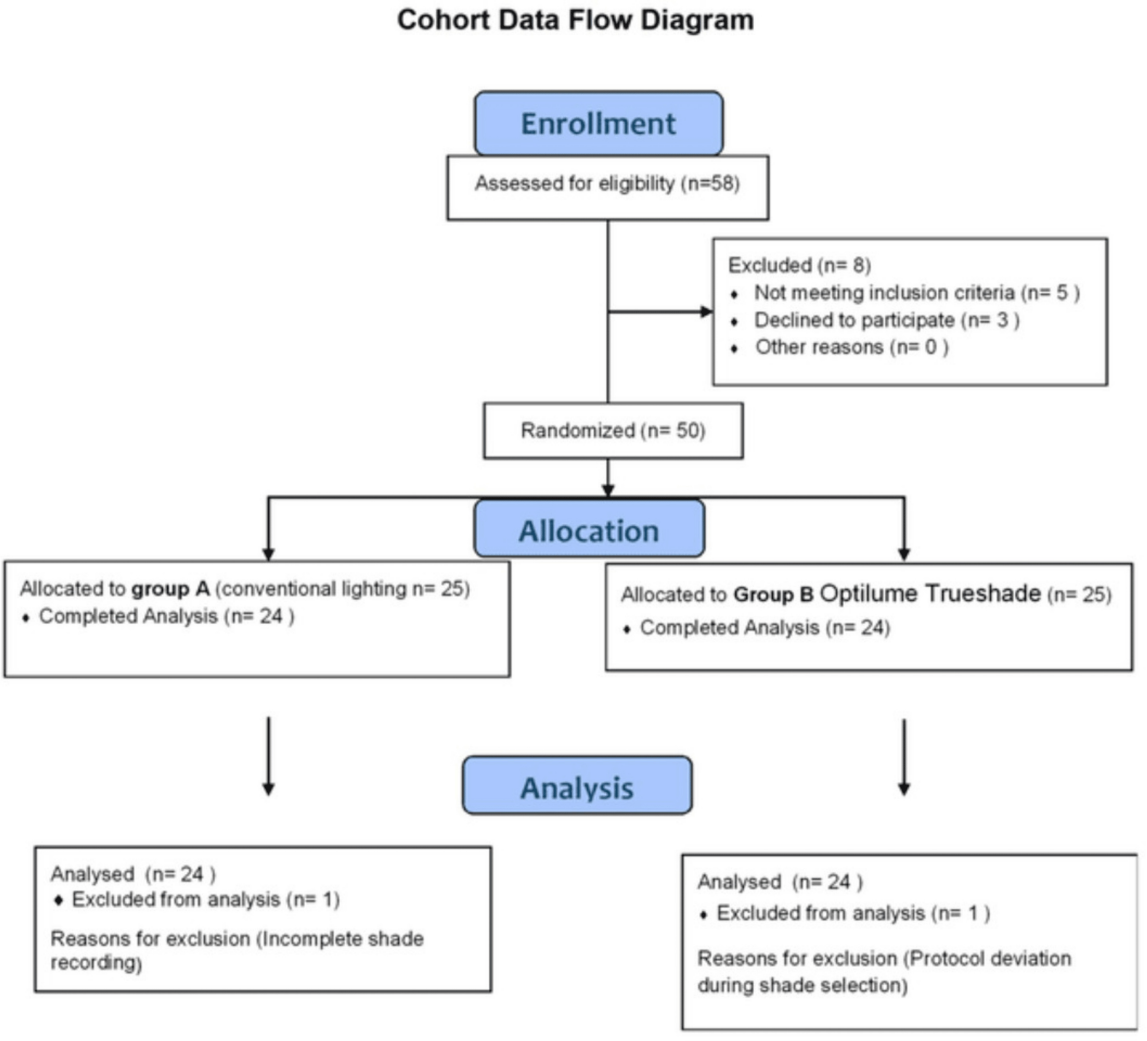
The participant recruitment and allocation process

A total of 50 participants (aged 18-40 years; mean age: 26.18 ± 5.92 years) were recruited. Participants were randomly allocated to two equal groups (n = 25 each): Group A: conventional operatory lighting; Group B: light-correcting device (Optilume Trueshade, ~5,500 K; Optident Dental Products, Ilkley, West Yorkshire) (Figure 1).

Inclusion criteria

The inclusion criteria were healthy adults aged 18-40 years, with intact, vital maxillary anterior teeth (Fédération Dentaire Internationale (FDI) #12-22), and no history of bleaching or restorative procedures on the anterior teeth.

Exclusion criteria

Exclusion criteria included teeth with restorations, discoloration (intrinsic or extrinsic), developmental defects or structural abnormalities, nonvital or endodontically treated teeth, and patients with conditions that affect color perception.

Data collection

Participants are randomized to Group A, Conventional Visual shade selection under standardized operatory/ambient conditions typical of clinical practice (dental unit/ambient lighting as per clinic norms), and Group B, Optilume Visual shade selection under the Optilume Trueshade device (~5,500 K; high-CRI daylight-simulating light-emitting diodes). Both groups used the VITAPAN Classical A1-D4 shade guide (VITA Zahnfabrik, Bad Säckingen, Germany), a widely validated visual shade-matching system in restorative dentistry [8,9]. Three restorative dentists undergo calibration, work independently, and are blinded to each other’s selections; analysts are blinded to group allocation. For a single reference tooth (randomly chosen from 12-22), the labial surface is conceptually segmented into cervical/middle/incisal thirds; the middle third is used for the definitive shade call due to lower gingival/incisal confounding.

Statistical analysis

Data were analyzed using IBM Statistical Package for the Social Sciences Statistics for Windows, version 26.0 (IBM Corp., Armonk, NY). Interexaminer reliability was assessed using Cronbach’s alpha. Differences in shade selection were analyzed using the Kruskal-Wallis test followed by Dunn-Bonferroni post hoc tests. Significance was considered at p ≤ 0.05.

## Results

The study included 50 participants with a mean age of 26.18 ± 5.92 years. The majority were male participants (n = 28, 56%), and most participants were within the 18-25-year age group (n = 22, 44%) (Table 1). The internal consistency of shade selection was excellent, yielding an overall Cronbach’s alpha of 0.94 and individual system scores of 0.91 for both VITAPAN Classical and Optilume Trueshade (Table 2). The cohort flowchart (Figure 1) illustrates participant recruitment, allocation, and analysis. Two participants (one from each group) were excluded from the final analysis due to incomplete data and protocol deviation.

**Table 1:** Sociodemographic characteristics of participants (n = 50)

Variable	Category	Frequency (n)	Percentage (%)
Age group	18-25 years	22	44%
	26-33 years	18	36%
	34-40 years	10	20%
Gender	Male	28	56%
	Female	22	44%
Education level	Undergraduate	30	60%
	Graduate	20	40%

**Table 2:** Reliability and internal consistency analysis

Assessment category	Cronbach’s alpha (α)	Number of items (n)	Percentage (%)	Interpretation
Total study reliability	0.94	24	100%	Excellent consistency
VITAPAN classical only	0.91	12	50%	High dependability
Optilume Trueshade only	0.91	12	50%	High dependability

While comparative testing indicated no statistically significant differences in shade selection for teeth 11, 12, and 22 across the six operator groups, a significant discrepancy was specifically observed for tooth 21, where the p value was 0.002, and the chi-square value was 19.07 (Table 3). Subsequent post hoc testing revealed that this variation was operator-specific rather than system-dependent, as VITAPAN Operator 1 was the only examiner to exhibit a significantly different shade-selection pattern compared with all other participants (Table 3). These findings suggest that while both the 5,500 K light-correcting device and conventional guides are highly reliable and produce stable outcomes for the majority of anterior teeth, individual human factors, such as physiological color perception, fatigue, and observation angles, remain the most significant variables in visual shade matching (Table 4).

**Table 3:** Comparison of shade selection by tooth (Kruskal-Wallis test) ^*^Statistically significant difference at p ≤ 0.05 FDI: Fédération Dentaire Internationale

Tooth position (FDI)	Chi-squared (χ^2^)	df	p value	Significance
Tooth 11	4.18	5	0.524	Not significant
Tooth 12	5.27	5	0.383	Not significant
Tooth 21	19.07	5	0.002*	Statistically significant
Tooth 22	7.39	5	0.193	Not significant

**Table 4:** Post-hoc analysis of significant variation (tooth 21) ^*^Statistically significant adjusted p values after Dunn-Bonferroni correction VP^*^: VITAPAN OT^**^: Optilume Trueshade

Comparison pair (tooth 21)	Test statistic	p value	Adjusted p value
VP^*^ operator 1 vs. VP operator 2	54.45	0.002	0.023^*^
VP operator 1 vs. VP operator 3	62.90	<0.001	0.004^*^
VP operator 1 vs. OT^**^ operator 1	57.08	0.001	0.014^*^
VP operator 1 vs. OT operator 2	57.76	0.001	0.012^*^
VP operator 1 vs. OT operator 3	56.71	0.001	0.015^*^

Although 50 participants were initially recruited and randomized, two datasets (one from each group) were excluded from the final analysis due to incomplete shade recordings and protocol deviations. Therefore, the final analyzed sample consisted of 48 participants. This adjustment explains the total number reflected in Table 2.

When comparing the two groups, both conventional lighting (Group A) and Optilume Trueshade (Group B) demonstrated comparable interexaminer reliability, with identical Cronbach’s alpha values (α = 0.91). No statistically significant differences were observed between the two groups for teeth 11, 12, and 22 (p > 0.05). Although a statistically significant difference was observed for tooth 21 (p = 0.002), post hoc analysis confirmed that this variation was attributable to a single operator rather than to lighting conditions. Overall, these findings indicate that both lighting conditions yield similar reliability outcomes, with no clear superiority of one method over the other. The absence of statistically significant intergroup differences suggests that environmental standardization alone may not fully eliminate operator-dependent variability in visual shade matching.

## Discussion

Achieving a lifelike color match in the anterior region remains a challenge because tooth appearance is heavily influenced by illumination and observer variability. This study compared the Optilume Trueshade light-correcting device (~5,500 K) with conventional operatory lighting using the VITAPAN Classical guide.

With a Cronbach's alpha of 0.91 for both systems, the results showed outstanding internal consistency. Both approaches are reliable for clinical shade selection, as indicated by the overall dependability score of 0.94. There were no statistically significant variations in the shades chosen by the three operators for the majority of the teeth tested (FDI 11, 12, and 22), suggesting that both the Optilume and VITAPAN systems function similarly under controlled conditions. For tooth 21, however, there was a notable difference (p = 0.002). Because it was primarily due to a single examiner, post hoc Dunn-Bonferroni testing showed that this variance was operator-specific rather than a defect in the shade guide system. This variety highlights the influence of human variables on esthetic results, such as weariness, visual adaptation, or positioning angles.

According to clinical data, standardized 5,500 K, high-CRI lighting is necessary to prevent metamerism and lessen these perceptual mistakes [10]. While digital tools such as spectrophotometers are available [11], they often do not fully agree with visual methods, making the optimization of visual workflows a clinically relevant and pragmatic strategy [12]. Consequently, while both systems are reliable, implementing light-correcting devices can help standardize the environment, especially where digital hardware is unavailable [9,13]. Standardized illumination ensures that minute color details are captured precisely and clearly, much like a high-definition lens [14].

The Kruskal-Wallis analysis revealed that for most shade groups (11, 12, and 22), there were no statistically significant differences among the six operator groups. This suggests that, in general, shade determination using both VITAPAN and Trueshade is consistent among different operators, regardless of their individual subjective judgment. Such consistency is important in restorative dentistry, where shade matching requires reproducibility to achieve optimal esthetic outcomes.

The present study showed that standardizing the lighting environment alone does not completely eliminate variability in visual shade matching. While both conventional lighting and the light-correcting device produced highly reliable outcomes, the persistence of operator-specific differences highlights that shade selection is not purely a technical process but also a perceptual one.

In practical clinical settings, dentists often assume that improving external conditions, such as using daylight-simulating devices, will automatically enhance accuracy. However, the findings of this study suggest that human visual perception remains the dominant influencing factor, even when environmental conditions are optimized, i.e., observer experience, eye fatigue, viewing angle, and cognitive interpretation of color.

Furthermore, the findings highlight that variability is more likely to appear in borderline or ambiguous shades, where subtle color differences are harder to distinguish. This may explain why significant variation was observed only in a specific tooth rather than across all samples. In such scenarios, clinicians should adopt a cautious approach, potentially combining multiple shade-selection methods or seeking consensus to improve accuracy.

Future research and practice should focus on several key areas. Operator calibration remains essential, as there is a clear need for strengthened training protocols and periodic calibration of clinicians to minimize observer-based discrepancies. In addition, future research should examine integrated or hybrid workflows that optimize visual shade matching alongside digital instruments, since visual and digital methods (such as intraoral scanners) often demonstrate relatively minimal concordance. Furthermore, incorporating these concepts into dental education may enhance clinical outcomes, as early exposure to light-correcting devices could improve shade-matching accuracy among dental students.

## Conclusions

Trueshade and VITAPAN both exhibit exceptional dependability and generally constant performance between operators. Shade 21 exhibited operator-specific variations, indicating a need for improved training procedures, even though most shade selections showed no discernible interoperator variability. However, for shade matching in restorative dentistry, both systems may be considered trustworthy instruments.
